# The “efficacy window” phenomenon of daridorexant in insomnia treatment: a dose-dependent time-course meta-analysis

**DOI:** 10.3389/fphar.2026.1847130

**Published:** 2026-06-30

**Authors:** Xiaorui Wang, Yixin Wang, Miaomiao Wang, Qiang Zheng, Mengyao Li, Yiqing Zheng, Yongli Gao, Wenliang Song, Jingru Bo, Huien Guo, Zikang Wang, Song Luo

**Affiliations:** 1 Department of Emergency, The Eighth People’s Hospital of Zhengzhou, The Mental Health Center of Zhengzhou, Zhengzhou, China; 2 Department of Neonatology, Bengbu First People’s Hospital, Bengbu, China; 3 Department of Neurology, The First Affiliated Hospital of Bengbu Medical University, Bengbu, China; 4 Medical College, Bengbu Medical University, Bengbu, China

**Keywords:** daridorexant, insomnia, meta-analysis, randomized controlled trial, systematic review

## Abstract

**Objective:**

This study aims to comprehensively analyze and evaluate the efficacy and safety of daridorexant in treating insomnia.

**Methods:**

This study conducted a systematic search across 7 electronic databases: PubMed, Web of Science, Cochrane Library, Embase, ClinicalTrials.gov, Scopus and the World Health Organization International Clinical Trials Registry Platform [http://www.who.int/ictrp/search/en/]). The review was registered (CRD420251174557). The effectiveness indicators included objective measures such as latency to persistent sleep (LPS) and wake time after sleep onset (WASO), as well as subjective measures including subjective latency to sleep onset (sLSO), subjective total sleep time (sTST), Visual analog scale (VAS), the Insomnia Daytime Symptoms and Impacts Questionnaire (IDSIQ) sleepiness domain score, and insomnia severity index (ISI). The safety indicators encompassed treatment-emergent adverse events (TEAEs), adverse events leading to premature termination, severe adverse events (SAEs), and adverse events of special interest (AESIs).

**Results:**

This study included a total of 5 articles, 6 RCTs, with a total of 2905 patients with insomnia. In terms of objective indicators, during the initial treatment stage (1–2 days), daridorexant was superior to the placebo; this advantage persisted at 1 month; and at 3 months, the 25 mg dose remained effective. In terms of subjective indicators, at 1 month, Daridorexant outperformed the placebo in most of the indicators; at 3 months, the 25 mg dose remained effective in sTST (SMD 0.25 [0.14, 0.36], *P* < 0.0001, I2 = 52%). In safety indicators, TEAEs, SAEs and adverse events leading to premature termination at each dose level were comparable to those of the placebo group. However, the combined effect indicated that the overall risk of TEAEs in daridorexant was higher (RR 1.15 [1.05, 1.25], *P* = 0.003, I^2^ = 0%). 25mg daridorexant significantly increased the risk of AESIs compared to placebo, while 10mg and 50 mg did not reach statistical significance, but overall still suggested an elevated risk (RR 4.00 [1.64, 9.76], *P* = 0.002, I^2^ = 0%).

**Conclusion:**

The efficacy of daridorexant in the treatment of insomnia is dose-dependent, with doses of 25 mg and 50 mg demonstrating significant improvements in both subjective and objective sleep metrics in the short term (1 month). Notably, the efficacy of the 25 mg dose is sustained in objective metrics over the medium term (3 months). Daridorexant overall performance is relatively safe, but the 25 mg dose may be associated with an increased risk of AESIs.

**Systematic Review Registration:**

Identifier, CRD420251174557.

## 1 Introduction

Insomnia refers to persistent difficulties with sleep onset or maintenance, often accompanied by concerns regarding sleep, dissatisfaction with sleep quality, or daytime functional impairment (Chinese Sleep Research Society, 2025). Insomnia is prevalent within the general population and frequently manifests as a chronic condition, persisting for several years. The most precise estimate of the prevalence of insomnia symptoms stands at 12.4%, with a self-reported prevalence of 16.3% based on the diagnostic criteria outlined in the Diagnostic and Statistical Manual of Mental Disorders (van Straten et al., 2025). The persistence rates of insomnia over a 1-year follow-up period are recorded at 86.0%, 72.4% over a 3-year follow-up, and 59.1% over a 5-year follow-up (Morin et al., 2020). Insomnia not only significantly impacts patients’ quality of life, cognitive function, and emotional wellbeing but is also closely linked to an elevated risk of cardiovascular diseases, metabolic syndrome, and accidents (Morin et al., 2022). Traditionally, benzodiazepine derivatives and Z-drugs have served as the mainstay of pharmacological treatment for insomnia (Krystal et al., 2023). However, concerns regarding the heightened risk of falls, residual effects the following morning, dose tolerance, dependence, and withdrawal symptoms (Wang et al., 2021; Yue et al., 2023), alongside their mechanism of action which involves widespread inhibition of the central nervous system, have sparked increased interest in alternative therapies. In recent years, orexin receptor antagonists (ORA) have emerged as a new class of medications with a unique mechanism of action, selectively blocking the orexin peptides in the hypothalamus that promote wakefulness, thereby facilitating more physiological sleep induction and maintenance (Sun et al., 2021). This development offers a novel option for the treatment of insomnia. The orexin receptor antagonists usually refer to single-target ones (OX1 or OX2), while dual orexin receptor antagonists (DORA) simultaneously block both receptors, resulting in better sleep assistance effects. Daridorexant, a second-generation DORA, has recently received approval from the U.S. FDA, the European Union, and the China National Medical Products Administration for the treatment of adult insomnia and is recommended for use in clinical guidelines (Riemann et al., 2023). However, daridorexant demonstrated varying degrees of efficacy and safety within the 5–50 mg dosage range. However, the optimal therapeutic window (the range between the minimum effective dose and the maximum tolerated dose) is still not clear. This study aims to quantitatively assess the therapeutic window of daridorexant through a dose-stratified meta-analysis, thereby providing evidence-based guidance for clinical dosage selection.

## 2 Materials and methods

### 2.1 Protocol and registration

This study adheres to the guidelines set forth in the Cochrane Collaboration Handbook and the Preferred Reporting Items for Systematic Reviews and Meta-Analyses (PRISMA) guidelines. It has been prospectively registered with PROSPERO (CRD420251174557).

### 2.2 Literature search and eligibility criteria

The inclusion criteria are established as follows: 1) Study type: Randomized controlled trial; 2) Participants: Patients diagnosed with primary insomnia in accordance with the Diagnostic and Statistical Manual of Mental Disorders, Fourth Edition, Text Revision (DSM-IV-TR) or the Diagnostic and Statistical Manual of Mental Disorders, Fifth Edition (DSM-V); 3) Intervention: Daridorexant; 4) Control: Placebo. The exclusion criteria are as follows: 1) Study type: Non-randomized controlled trial, review, conference, protocol, and case report; 2) Participants: Patients under 18 years of age, as well as patients with comorbid physical and mental disorders; 3) Intervention: Other treatments.

### 2.3 Outcome measures

Efficacy measures encompass both objective and subjective outcomes pertaining to sleep maintenance and onset. The objective sleep-related outcomes were assessed using polysomnography, which included measurements of latency to persistent sleep (LPS) and wake time after sleep onset (WASO) during the initial sleep period (Days 1 and 2), as well as at 1 month and 3 months. Subjective sleep outcomes were assessed via an electronic morning sleep diary, which included the subjective latency to sleep onset (sLSO), Visual Analog Scale (VAS), and Insomnia Severity Index (ISI) (Morin et la., 2011) at month 1, as well as the subjective total sleep time (sTST) and the Insomnia Daytime Symptoms and Impacts Questionnaire (IDSIQ) sleepiness domain score (Hudgens et al., 2021) at months 1 and 3. Among them, IDSIQ sleepiness domain score were the mean of the seven entries in the week before polysomnography recording nights (Mignot et al., 2022). The mean of the weekly entries (of at least 3 days during a week) for each sleep diary entry was used to calculate the weekly average score at baseline and Week 4. If weekly data were available from less than 3 days, the mean value of that week was considered missing (Uchimura et al., 2024).

Safety endpoints encompass treatment-emergent adverse events (TEAEs), adverse events leading to premature termination, serious adverse events (SAEs), and adjudicated potential adverse events of special interest (AESIs). AESIs were predefined as preferred term-coded AEs related to narcolepsylike events, complex sleep behavior events, and suicidal thoughts and/or behaviors (Uchimura et al., 2024; Mignot et al., 2022).

### 2.4 Search strategy

Two independent researchers conducted a comprehensive search of seven databases: PubMed, Embase, Cochrane Library, Web of Science, Scopus, ClinicalTrials.gov, and the World Health Organization International Clinical Trials Registry Platform [http://www.who.int/ictrp/search/en/], covering the period from the inception of the databases to 19 October 2025. (Note: For PubMed and ClinicalTrials.gov, due to the interruption of U.S. federal government funding, the search concluded on 1 October 2025.). The search terms were “Daridorexant” and “insomnia”. The search methods and strategies are elaborated in Supplementary Table S1 **.**

### 2.5 Study selection and data collection

The two authors conducted an independent review of all records obtained from the electronic databases in accordance with the aforementioned eligibility criteria. Duplicate entries and articles consisting solely of abstracts were excluded. Any disagreements between the two authors were addressed through discussion or resolved by a third author who was not involved in the data collection process. The following details will be extracted: author, publication year, clinical trial registration number, study design, age range, gender distribution, intervention method, duration, and follow-up period.

### 2.6 Risk of bias and quality of evidence

The Cochrane Risk of Bias Assessment Tool, RoB 1.0, was employed to assess the methodological quality of the included randomized controlled trials. For each domain, the risk of bias was classified as “low risk,” “high risk,” or “unclear risk.” Subsequently, an overall judgment regarding the risk of bias was made for each study based on the evaluation results of each domain. All assessments were conducted using Review Manager 5.3.

### 2.7 Data analysis

In this study, all data analyses and the creation of forest plots were conducted using RevMan 5.3 software. The chi-square test (*P*-value) and the I^2^ test were employed to evaluate heterogeneity. Dose stratification and sensitivity analyses were performed to assess the impact of reuse of shared placebo data (due to the original design) in multiple-dose studies. When the result of the I^2^ test was less than 50% and *P* > 0.05, indicating the absence of significant heterogeneity, a fixed-effect model was utilized. Conversely, when the I^2^test result exceeded 50% and *P* ≤ 0.05, indicating significant heterogeneity, a random-effect model was implemented, and sensitivity analysis was performed. The standardized mean differences (SMD) along with its 95% confidence interval (CI) served as summary statistics for continuous variables. For dichotomous variables, the risk ratio (RR) with a 95% CI was employed as the summary statistic, with statistical significance established at *P* < 0.05.

## 3 Results

### 3.1 Search results and baseline characteristics

**FIGURE 1:**
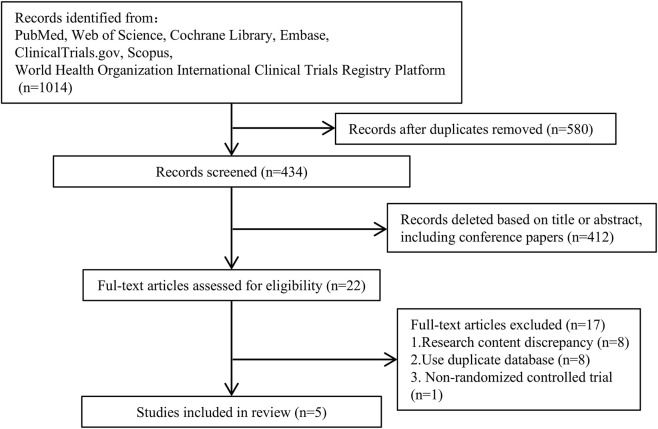
PRISMA flowchart of the screening and selection of studies.

A total of 1,014 results were retrieved (194 from Web of Science, 125 from PubMed, 222 from Scopus, 112 from the Cochrane Library, 319 from Embase, 26 from the World Health Organization International Clinical Trials Registry Platform, and 16 from ClinicalTrials.gov). After removing duplicate results, 434 articles met the title and abstract screening criteria. Following the title and abstract screening, only 22 studies were selected for full-text review. Finally, 5 articles were included after screening (Figure 1). Since Mignot et al. (2022) contained two RCT studies, a total of 6 RCT studies were ultimately included. Apart from Uchimura et al. (2024) from Japan and Huang et al. (2025) from China, the remaining studies were mainly from Europe and the United States. This study included 2,905 insomnia patients, of whom 1,885 (64.8%) were female. Study durations ranged from 1 to 3 months, with follow-up periods of 1 month Table. 1 provides a summary of the baseline characteristics of the included studies.

**TABLE 1:** Summary of characteristics included in the study.

Study ID	Registration number	Study design	Age (years)	Female (n, %)	Treatment groups	Duration	Follow-up
Huang et al. (2025)	NCT06010693	RCT	18–75	150 (72.8%)	Daridorexant 50 mg vs. placebo	1 month	4 weeks
Uchimura et al. (2024)	jRCT2031200452	RCT	≥18	242 (49.6%)	Daridorexant 50 mg,25 mg vs. placebo	28 days	30 days
Dauvilliers et al. (2020)	NCT02839200	RCT	18–64	192 (64.2%)	Daridorexant 50 mg,25 mg,10 mg,5 mg vs. placebo	30 days	30 days
Mignot et al. (2022) study1	NCT03545191	RCT	≥18	624 (67.1%)	Daridorexant 50 mg,25 mg vs. placebo	12 weeks	30 days
Mignot et al. (2022) study2	NCT03575104	RCT	≥18	638 (69.0%)	Daridorexant 25 mg,10 mg vs. placebo	12 weeks	30 days
Zammit et al. (2020)	NCT02841709	RCT	≥65	39 (67.0%)	Daridorexant 50 mg,25 mg,10 mg,5 mg vs. placebo	2 days	30 days

RCT: randomized controlled trial.

### 3.2 Quality assessment

The Cochrane Risk of Bias tool (RoB 1.0) was utilized to evaluate seven domains of bias in each study: generation of random sequences, allocation concealment, blinding of participants and personnel, blinding of outcome assessors, incomplete outcome data, selective reporting, and other biases. The figure illustrates that the majority of studies exhibited a ‘low risk’ of bias in the aforementioned key domains, indicating an overall low risk of bias. A detailed distribution of the risk of bias is presented in Supplementary Figure S1 **.**

### 3.3 Efficacy outcomes

#### 3.3.1 Objective indicators

**FIGURE 2:**
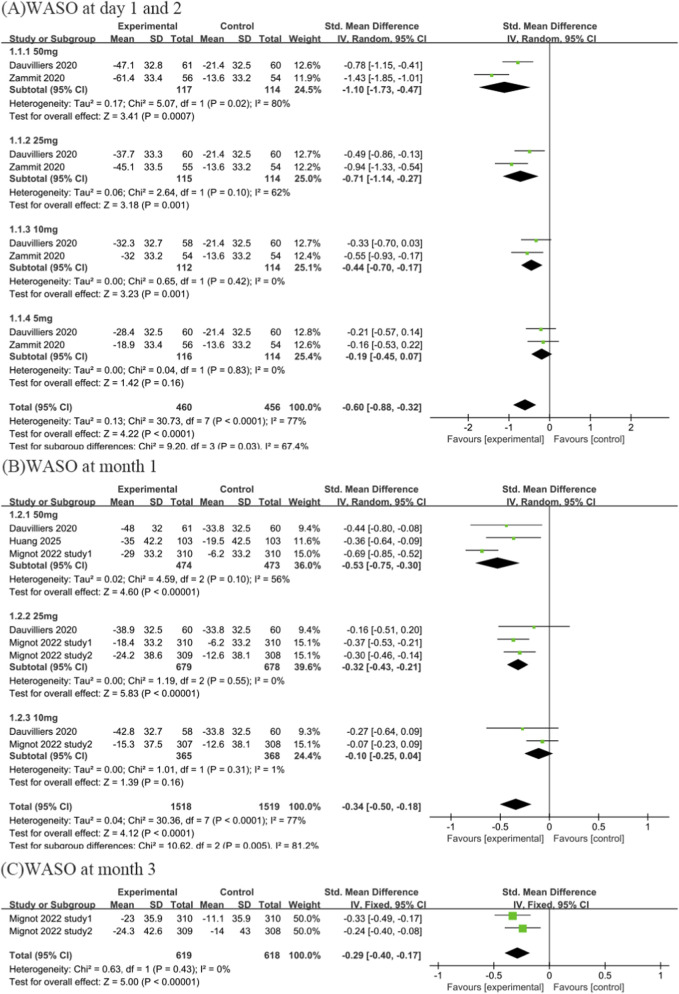
The pooled SMD of efficacy outcomes in different doses compared with placebo. **(A)** WASO at day 1 and 2. **(B)** WASO at month 1. **(C)** WASO at month 3.

WASO:A total of 5 research reports described the changes in WASO relative to the baseline. The results showed that compared with placebo, the daridorexant group had significantly lower WASO at day 1 and 2 (SMD -0.60 [-0.88, −0.32], *P* < 0.0001), WASO at month 1 (SMD -0.34 [-0.50, −0.18], *P* < 0.0001), and WASO at month 3 (SMD -0.29 [-0.40, −0.17], *P* < 0.00001) compared to the baseline (Figure 2). WASO at day 1 and 2 (I^2^ = 77%, *P* < 0.0001) and WASO at month 1 (I^2^ = 77%, *P* < 0.0001) had heterogeneity. A sensitivity analysis by sequentially excluding each study showed that the direction and statistical significance of the combined effect size did not change, suggesting that the main results were robust.

**FIGURE 3:**
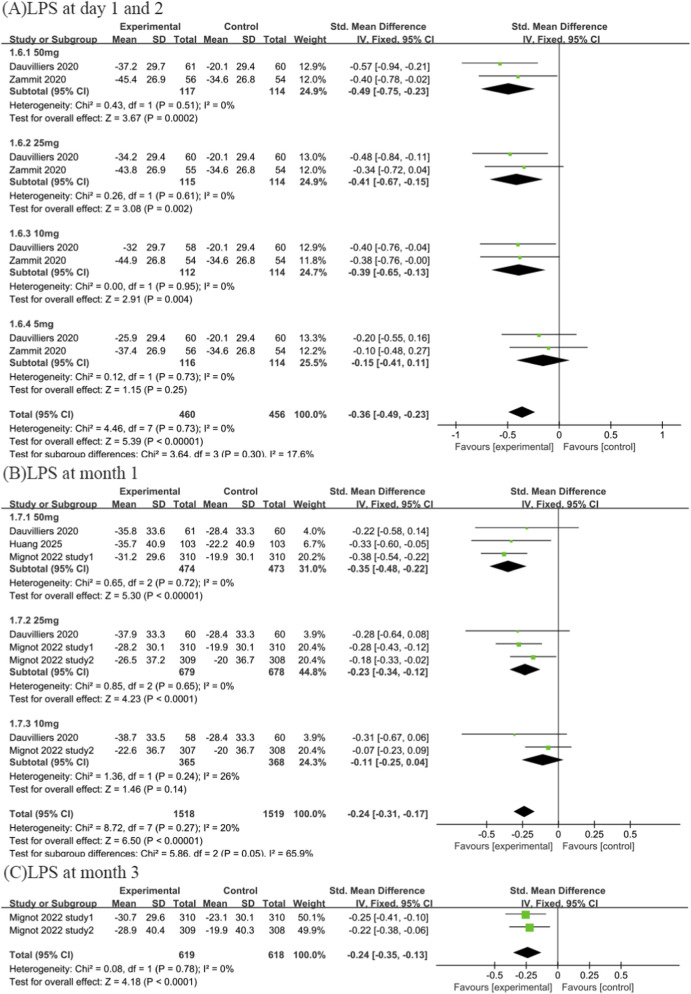
The pooled SMD of efficacy outcomes in different doses compared with placebo. **(A)** LPS at day 1 and 2. **(B)** LPS at month 1. **(C)** LPS at month 3.

LPS:A total of 5 studies reported changes in LPS relative to baseline. The results indicated that compared with placebo, the daridorexant group showed a significant reduction in LPS at day 1 and 2 (SMD -0.36 [-0.49, −0.23], *P* < 0.00001, I^2^ = 0%) and LPS at month 1 (SMD -0.24 [-0.31, −0.17], *P* < 0.00001, I^2^ = 20%) relative to baseline. At month 3, the 25 mg dose (SMD -0.24 [-0.35, −0.13], *P* < 0.0001, I^2^ = 0%) still demonstrated sustained significant improvement (Figure 3).

#### 3.3.2 Subjective indicators

sLSO: A total of 2 reports on sLSO changes relative to baseline were documented. Compared with placebo, the daridorexant group showed a significant reduction in sLSO at month 1 (SMD -0.31 [-0.44, −0.17], *P* < 0.00001, I^2^ = 26%), Supplementary Figure S2 **.**

sTST: A total of 5 reports concerning changes in sTST relative to the baseline were documented. The results indicated that the daridorexant group experienced a significant increase in sTST at month 1 compared to the placebo group (0.31 [0.25, 0.38], *P* < 0.00001, I^2^ = 11%). Additionally, the sTST at month 3 with 25 mg (SMD 0.25 [0.14, 0.36], *P* < 0.0001, I^2^ = 52%) continued to demonstrate a significant improvement, Supplementary Figure S3 **.**

VAS: A total of 4 studies reported VAS scores at 4 weeks. The results indicated that the daridorexant groups had higher scores than the placebo group at 4 weeks (*P* < 0.05). In terms of sleep quality, sleep depth, daytime alertness, and ability to function, both the 50 mg and 25 mg groups outperformed the placebo group (*P* < 0.05). The 10 mg group demonstrated no significant difference from the placebo group regarding sleep quality, aytime alertness, and ability to function (*P* > 0.05) Supplementary Figures S4-S7 **.**

IDSIQ Sleepiness Domain Score: A total of 2 studies reported the IDSIQ sleepiness domain score. The results showed that, compared with placebo, 25 mg of daridorexant significantly reduced the IDSIQ sleepiness domain score at 1 month (SMD -0.15 [-0.26, −0.04], P = 0.009, I^2^ = 0%) and at 3 months (SMD -0.18 [-0.29, −0.07], P = 0.001, I^2^ = 0%), as shown in Supplementary Figure S8 **.**

ISI: A total of 3 reports on ISI at 4 weeks were reported. The results showed that daridorexant 50mg and 25 mg had lower ISI than the placebo group at 4 weeks (*P* < 0.05), while there was no statistically significant difference between daridorexant 10 mg and placebo. After combining the effect sizes, ISI was significantly lower than that of the placebo (SMD -0.21 [-0.28, −0.13], *P* < 0.00001, I^2^ = 5%). Supplementary Figure S9 **.**

### 3.4 Safety outcomes

**FIGURE 4:**
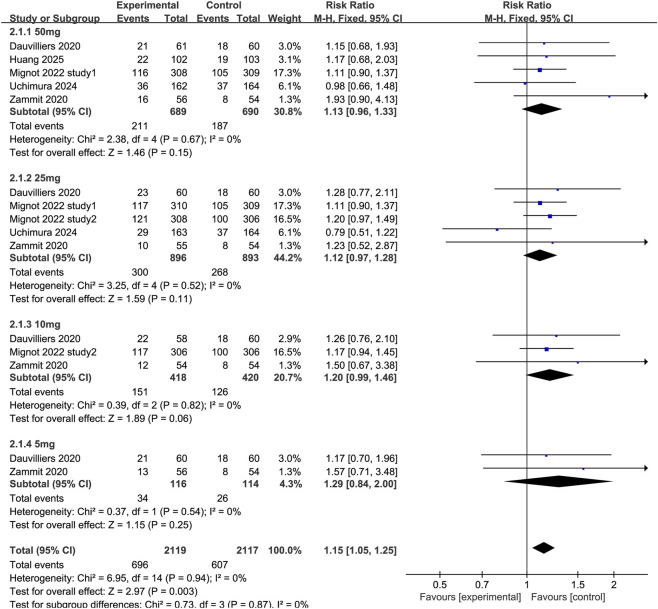
The pooled RR of treatment-emergent adverse events. CI = confidence interval.

TEAEs: A total of 6 reports on TEAEs were included in this analysis. The results indicated no statistically significant difference between daridorexant and placebo regarding adverse events (*P* > 0.05). Upon pooling the effect sizes, daridorexant was found to be inferior to placebo (RR 1.15 [1.05, 1.25], *P* = 0.003, I^2^ = 0%)(Figure 4). The common adverse events observed included nasopharyngitis (RR 1.10 [0.83, 1.47], *P* = 0.49, I^2^ = 23%), headache (RR 1.60 [1.15, 2.23], *P* = 0.005, I^2^ = 0%), somnolence (RR 1.79 [1.20, 2.69], *P* = 0.005, I^2^ = 0%), fatigue (RR 2.01 [1.21, 3.36], *P* = 0.007, I^2^ = 2%), and dizziness (RR 1.65 [0.93, 2.93], *P* = 0.09, I^2^ = 0%). Headache, somnolence, and fatigue were found to be inferior to placebo, while nasopharyngitis and dizziness did not demonstrate a statistically significant difference compared to placebo Supplementary Figures S10-14, Table S1.

SAEs: A total of 5 studies reported SAEs, and the results indicated that, compared with placebo, daridorexant showed no statistically significant difference in the incidence of serious adverse events (RR 0.73 [0.41, 1.32], *P* = 0.30, I^2^ = 0%), Supplementary Figure S15 **.**

Adverse events leading to premature termination: A total of 6 studies reported adverse events leading to premature termination. The results indicated that, compared with placebo, daridorexant showed no statistically significant difference in the incidence of adverse events leading to premature termination (RR 0.85 [0.54, 1.34], *P* = 0.48, I^2^ = 0%), Supplementary Figure S16 **.**

**FIGURE 5:**
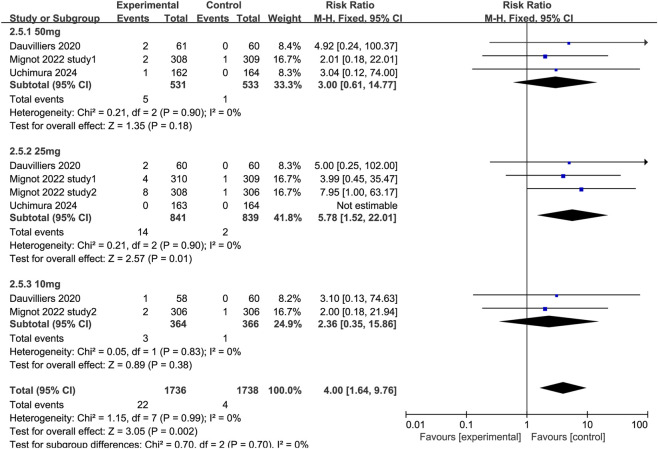
The pooled RR of Adjudicated adverse events of special interest. CI = confidence interval.

AESIs: 4 studies reported AESIs, with results indicating that daridorexant was inferior to placebo in terms of AESI incidence (RR 4.00 [1.64, 9.76], P = 0.002, I^2^ = 0%), as shown in Figure 5. Sleep paralysis analysis showed that the difference between daridorexant and placebo was not statistically significant. (RR 3.50 [0.73, 16.83], P = 0.12, I^2^ = 0%), Supplementary Figure S17 **.**

## 4 Discussion

This study conducted a systematic evaluation of the time-dependent efficacy and safety characteristics of various doses of daridorexant for the treatment of insomnia, based on a meta-analysis of existing randomized controlled trials. The findings revealed distinct dose-response and time-response relationships, and identified potential risk signals at certain doses, thereby offering critical evidence for clinical decision-making.

This study confirms that daridorexant effectively improves LPS and WASO, consistent with the core pharmacological mechanism of DORAs in suppressing central arousal signals (Muehlan et al., 2023). The results demonstrate a clear dose-response gradient (50 mg > 25 mg > 10 mg ≈ 5 mg) and time-dependent efficacy of daridorexant. Higher doses (25 mg and 50 mg) exhibited significant therapeutic effects early in treatment (days 1–2), and this benefit persisted at the 1-month assessment. Moreover, the 25 mg dose maintained sustained efficacy at the 3-month time point. Data for the 50 mg dose at 3 months could not be further analyzed due to limited sample size or study constraints. Jiang et al. (2023) reported that 50 mg daridorexant outperformed placebo in WASO, sTST, LPS, and IDSIQ sleepiness domain score at 3 months, however, the analysis of these endpoints was based on only one study, which somewhat limits the strength of the evidence. An extension trial found (NCT03679884) the use of daridorexant (up to 12 months) to be generally safe and well tolerated. The sustained improvement in sleep and daytime functional status with daridorexant at a dose of 50 mg supports its use for the long-term treatment of insomnia, and there are no safety concerns (Kunz et al., 2023). It is noteworthy that the 10 mg dose improved sTST in the short term but did not differ significantly from placebo in objective measures and overall efficacy, suggesting its therapeutic effect may be weak or non-sustained. Furthermore, the 5 mg dosage of daridorexant showed no significant difference in both subjective and objective indicators compared to the placebo, suggesting that 5 mg might be an ineffective dose. Using linear regression and logistic regression, Luyet et al. (2024) identified a clear dose–response relationship for daridorexant efficacy within the 5–50 mg dose range, with higher doses corresponding to higher responder rates, which aligns with the findings of the present study. This study is the first to incorporate data from China and Japan. Compared with previous meta-analyses predominantly based on European and American populations (Jiang et al., 2023), the present work expands the evidence base to Asia, reflecting an evolution in sleep medicine research towards greater inclusivity and representativeness. It also provides direct support for the clinical application of this drug in Asian populations.

Current pharmacological treatments for insomnia can be categorized into γ-aminobutyric acid (GABA) modulators (including benzodiazepines and benzodiazepine receptor agonists [Z-drugs]), melatonin receptor agonists (MRAs), sedating antidepressants, and ORAs (Krystal et al., 2023). Compared with traditional GABAergic medications, daridorexant exhibits a distinct profile of advantages and limitations. A network meta-analysis based on 69 double-blind studies involving 17,319 patients and 20 pharmacological agents concluded that ORAs represent the preferred recommendation for sleep-maintenance insomnia, demonstrating significantly greater efficacy than other drug classes such as Z-drugs and MRAs. Notably, daridorexant achieved the highest surface under the cumulative ranking curve (SUCRA) value (0.80) for reducing sleep onset latency, second highest SUCRA value in WASO (0.86). suggesting a potential advantage in facilitating sleep initiation and maintenance (Yue et al., 2023). This finding aligns with the results of the present study, in which daridorexant showed superior improvement in LPS and sLSO compared to placebo. However, a network meta-analysis encompassing 170 trials (36 interventions, 47,950 participants) found that, in direct comparisons, short-acting benzodiazepines demonstrated superior efficacy to daridorexant and lemborexant after 4 weeks of treatment (De Crescenzo et al., 2022). This suggests that conventional agents may retain an advantage in terms of short-term absolute efficacy. In other words, while daridorexant is effective, it may not represent the “most potent” option in short-term head-to-head comparisons. Regarding the other two commercially available DORA agents (suvorexant and lemborexant), daridorexant 50 mg demonstrated superiority over both daridorexant 25 mg and lemborexant 5 mg in improving sTST during the first month, indicating that daridorexant 50 mg has the greatest impact on sTST within this initial period (Kishi et al., 2025). This finding aligns with the results of the present study, which showed that daridorexant 50 mg provided superior improvement in objective sleep measures. It is projected that daridorexant 50 mg will be particularly beneficial in addressing difficulties with sleep maintenance. An indirect comparison by Citrome et al. (2023) revealed that lemborexant demonstrated an advantage in efficacy (number needed to treat,NNT), whereas daridorexant exhibited an advantage in tolerability (number needed to harm, NNH), particularly regarding the risk of somnolence. The overall benefit-risk ratios (likelihood to be helped or harmed,LHH) for both agents were favorable but distinct in profile: lemborexant tended toward “greater potency,” while daridorexant leaned toward “better tolerability.” However, potential differences in specific duration of action and the degree of improvement in certain subjective measures may exist, which warrants further investigation.

This study confirms that daridorexant is generally well-tolerated. No statistically significant differences were observed between any dose group (5mg–50 mg) and the placebo group in terms of TEATs, SAEs, or adverse events leading to premature termination. Luyet et al. (2024) reported that the incidence of total adverse events plateaued at 10–25 mg, with no increased risk observed at 50 mg, which is consistent with the findings of the present study. The top 5 adverse events in this study were nasopharyngitis, headache, somnolence, fatigue, and dizziness. These adverse reactions were relatively mild and occurred at a low frequency (Supplementary Figures S10-14). This overall safety profile is similar to that of Lemborexant (Khan et al., 2025). Studies have shown that DORAs offer a superior safety profile compared to GABAergic agents, particularly in reducing the risk of falls/fractures (Matsumoto et al., 2023). Driving simulation studies further confirmed that daridorexant has no significant impact on psychomotor performance (Muehlan et al., 2022). Muehlan et al. (2020) reported minimal next-day residual effects with daridorexant. Its effects dissipated approximately 8 h after morning administration, and no residual effects (assessed both objectively and subjectively) were detected the following morning after an evening dose of 25 mg. This contrasts with the longer half-lives of lemborexant (17–19 h) and suvorexant (12 h), suggesting a potential safety/tolerability advantage for daridorexant (5.9–8.8 h) (Muehlan et al., 2018). This profile makes daridorexant an attractive option for individuals requiring a high degree of daytime alertness. A secondary analysis of an RCT (NCT03545191) found that patients aged ≥65 years did not exhibit an increased risk of adverse events or residual effects the following morning after taking 50 mg of daridorexant at night. This suggests that dose reduction may not be necessary for older adults when using daridorexant (Fietze et al., 2022). However, after pooling effect sizes across TEAEs, patients in the daridorexant group demonstrated a 15% higher overall likelihood of achieving a treatment response compared to the placebo group. Subgroup analyses revealed that daridorexant differed from placebo in specific adverse events. For instance, while daridorexant 10 mg was inferior to placebo for nasopharyngitis, the difference became statistically insignificant after effect size pooling. At this juncture, the methodological strengths of meta-analysis—enhancing power and precision—prove optimal for revealing such small yet genuine effects. This represents not a contradiction or error, but a higher-level evidence synthesis process that yields more reliable safety conclusions than any single study. Additionally, analysis of AESIs revealed a significantly higher risk in the 25 mg dose group compared to placebo. The 50 mg dose demonstrated robust efficacy within 1 month without showing an increased risk of AESIs. However, in the combined analysis across all doses, the risk of AESIs in the daridorexant group remained significantly higher than placebo. This suggests that AESI risk is a concern requiring attention for daridorexant, with the 25 mg dose potentially representing a specific range where this risk is concentrated. Although McIntyre et al. (2025) reported that suvorexant, lemborexant, and daridorexant were associated with a significantly lower odds of completed suicide compared to trazodone, and the present study found no statistically significant difference between daridorexant (50 mg and 25 mg) and placebo in the incidence of sleep paralysis (Supplementary Figure S17), this dose-specific risk signal warrants clinical vigilance. It reveals a non-linear dose-response relationship. We hypothesize that a dosage of 25 mg may fall within a sensitive range that sustains long-term efficacy while potentially placing patients in a critical state of sleep-wake regulation, thereby increasing their susceptibility to perceiving or reporting mechanism-related events such as sleep paralysis. In contrast, the more profound antagonistic effect of 50 mg might diminish the awareness of such borderline states, which necessitates further validation in future studies. In addition, it is also possible that small risks were detected due to the largest sample size and longest follow-up at 25mg, lack of long-term data at 50mg, and insufficient exposure at 10 mg. For the two key safety endpoints: SAEs and adverse events leading to premature termination, all active dose groups (10mg, 25mg, 50 mg) showed no significant difference compared to placebo. This strongly suggests that daridorexant treatment does not pose unacceptable serious safety risks, and the severity and nature of its adverse events remain within tolerable limits for patients, insufficient to cause a significant increase in treatment discontinuation rates. Additionally, Mignot et al. (2022) found that daridorexant did not exhibit the dependence, tolerance, or next-day carryover effects commonly associated with traditional benzodiazepines/Z-drugs during long-term use, offering an important new option for patients requiring prolonged treatment. Whether used as initial therapy, as a switch, or as an adjunct, daridorexant has demonstrated efficacy and safety in real-world chronic insomnia disorder treatment without serious treatment-related adverse events (Fernandes et al., 2024). However, some studies have reported higher discontinuation rates for DORA compared to benzodiazepines, potentially associated with older patient age (Kurihara et al., 2026). Further studies have confirmed that daridorexant not only effectively alleviates insomnia symptoms but also reduces comorbid depression, anxiety, manic symptoms, and suicide risk in patients with insomnia accompanied by depression/anxiety. These therapeutic benefits remained stable over a 3-month continuous treatment period (Palagini et al., 2024). A post-hoc analysis of daridorexant in patients with untreated mild obstructive sleep apnea and comorbid insomnia found that, compared with placebo, daridorexant 50 mg improved all sleep parameters over time and was well-tolerated (Lettieri et al., 2026). These findings suggest that daridorexant holds promise as a novel therapeutic approach for a range of psychiatric disorders and psychosomatic conditions beyond insomnia.

Limitations of the Study:First, the follow-up duration in most included studies was limited to 1–3 months, and the lack of longer-term data restricts conclusions regarding sustained efficacy and long-term safety. Second, heterogeneity may exist in the definitions and measurement tools used for subjective outcomes across the studies. Third, subgroup analysis based on specific insomnia subtypes (e.g., sleep onset vs. sleep maintenance insomnia) was not performed. Finally, AESIs were not explored in detail. Further research is needed to clarify the risk profile associated with the 25 mg dose.

## 5 Conclusion

The efficacy of daridorexant in treating insomnia exhibits dose- and time-dependent characteristics. In clinical practice, daridorexant 50 mg may be prioritized for its rapid onset of action; for long-term maintenance, 25 mg can be considered, although this dosage is associated with an elevated risk of AESIs, indicating that the benefits and risks must be carefully evaluated in clinical use.

## Data Availability

The original contributions presented in the study are included in the article/Supplementary Material, further inquiries can be directed to the corresponding author.
