# Supplementary material for: The “efficacy window” phenomenon of daridorexant in insomnia treatment: a dose-dependent time-course meta-analysis
Source: Front Pharmacol. 2026 Jun 30;17:1847130. doi: 10.3389/fphar.2026.1847130 (PMC13364843; doi:10.3389/fphar.2026.1847130)
Supplement: Supplementary file 1 [file DataSheet1.docx]

**Supplementary Materials**

**Table S1 Search Method**

| **Set** | **Pubmed** | **Result** |
| --- | --- | --- |
| #1 | "Sleep Initiation and Maintenance Disorders"[Mesh] | 20637 |
| #2 | ((((((((((((((DIMS )) OR (Disorders of Initiating and Maintaining Sleep)) OR (Sleeplessness)) OR (Insomnia*)) OR (Chronic Insomnia)) OR (Early Awakening)) OR (Awakening, Early)) OR (Nonorganic Insomnia)) OR (Primary Insomnia)) OR (Psychophysiological Insomnia)) OR (Rebound Insomnia)) OR (Secondary Insomnia)) OR (Sleep Initiation*)) OR (Dysfunction*, Sleep Initiation)) OR (Transient Insomnia) | 49571 |
| #3 | #1OR#2 | 49571 |
| #4 | "daridorexant" [Supplementary Concept] | 66 |
| #5 | (((((S)-(2-(5-chloro-4-methyl-1H-benzo(d)imidazol-2-yl)-2-methylpyrrolidin-1-yl) (5 methoxy-2-(2H-1,2,3-triazol-2-yl)phenyl)methanone)) OR (methanone, ((2S)-2-(6-chloro-7-methyl-1H-benzimidazol-2-yl)-2-methyl-1-pyrrolidinyl)(5-methoxy-2-(2H-1,2,3-traizol-2-yl)phenyl)-)) OR (nemorexant)) OR (ACT-541468) | 146 |
| #6 | #4OR#5 | 146 |
| #7 | #3AND#6 | 125 |
| **Set** | **Web of Science** | **Result** |
| #1 | ((((TS=(Daridorexant)) OR TS=(((S)-(2-(5-chloro-4-methyl-1H-benzo(d)imidazol-2-yl)-2-methylpyrrolidin-1-yl) (5 methoxy-2-(2H-1,2,3-triazol-2-yl)phenyl)methanone))) OR TS=(methanone, ((2S)-2-(6-chloro-7-methyl-1H-benzimidazol-2-yl)-2-methyl-1-pyrrolidinyl)(5-methoxy-2-(2H-1,2,3-traizol-2-yl)phenyl)-)) OR TS=(nemorexant)) OR TS=(ACT-541468) | 228 |
| #2 | (((((((((((((((TS=(Sleep Initiation and Maintenance Disorders)) OR TS=(DIMS)) OR TS=(Disorders of Initiating and Maintaining Sleep)) OR TS=(Sleeplessness)) OR TS=(Insomnia*)) OR TS=(Chronic Insomnia)) OR TS=(Early Awakening)) OR TS=(Awakening, Early)) OR TS=(Nonorganic Insomnia)) OR TS=(Primary Insomnia)) OR TS=(Psychophysiological Insomnia)) OR TS=(Rebound Insomnia)) OR TS=(Secondary Insomnia)) OR TS=(Sleep Initiation*)) OR TS=(Dysfunction*, Sleep Initiation)) OR TS=(Transient Insomnia) | 131039 |
| #3 | #1 and #2 | 194 |
| **Set** | **Cochrane** | **Result** |
| #1 | MeSH descriptor: [Sleep Initiation and Maintenance Disorders] explode all trees | 4076 |
| #2 | (DIMS or Disorders of Initiating and Maintaining Sleep or Sleeplessness or Insomnia Disorder or Insomnia Disorders or Insomnia or Insomnias or Chronic Insomnia or Insomnia, Chronic or Early Awakening or Awakening, Early or Nonorganic Insomnia or Insomnia, Nonorganic or Primary Insomnia or Insomnia, Primary or Psychophysiological Insomnia or Insomnia, Psychophysiological or Rebound Insomnia or Insomnia, Rebound or Secondary Insomnia or Insomnia, Secondary or Sleep Initiation Dysfunction or Dysfunction, Sleep Initiation or Dysfunctions, Sleep Initiation or Sleep Initiation Dysfunctions or Transient Insomnia or Insomnia, Transient):ti,ab,kw | 18656 |
| #3 | #1 or #2 | 19236 |
| #4 | (daridorexant):ti,ab,kw | 104 |
| #5 | (nemorexant or ACT-541468):ti,ab,kw | 56 |
| #6 | #4 or #5 | 145 |
| #7 | #3 and #6 | 112 |
| **Set** | **Embase** | **Result** |
| #1 | sleep disorder'/exp | 405989 |
| #2 | sleep initiation and maintenance disorders':ti,ab,kw OR 'dims':ti,ab,kw OR 'disorders of initiating and maintaining sleep':ti,ab,kw OR 'sleeplessness':ti,ab,kw OR 'insomnia*':ti,ab,kw OR 'chronic insomnia':ti,ab,kw OR 'early awakening':ti,ab,kw OR 'awakening, early':ti,ab,kw OR 'nonorganic insomnia':ti,ab,kw OR 'primary insomnia':ti,ab,kw OR 'psychophysiological insomnia':ti,ab,kw OR 'rebound insomnia':ti,ab,kw OR 'secondary insomnia':ti,ab,kw OR 'sleep initiation*':ti,ab,kw OR 'dysfunction*, sleep initiation':ti,ab,kw OR 'transient insomnia':ti,ab,kw | 66653 |
| #3 | #1 or #2 | 414445 |
| #4 | daridorexant'/exp | 383 |
| #5 | ((s)-(2-(5-chloro-4-methyl-1h-benzo(d)imidazol-2-yl)-2-methylpyrrolidin-1-yl) (5 methoxy-2-(2h-1,2,3-triazol-2-yl)phenyl)methanone)':ti,ab,kw OR 'methanone, ((2s)-2-(6-chloro-7-methyl-1h-benzimidazol-2-yl)-2-methyl-1-pyrrolidinyl)(5-methoxy-2-(2h-1,2,3-traizol-2-yl)phenyl)-':ti,ab,kw OR 'dnemorexant':ti,ab,kw OR 'act-541468':ti,ab,kw | 51 |
| #6 | #4 or #5 | 387 |
| #7 | #3 and #6 | 319 |
| **set** | **ClinicalTrials.gov [http://clinicaltrials.gov/]** | **Result** |
| #1 | Sleep Initiation and Maintenance Disorders AND daridorexant | 16 |
| **set** | **the World Health Organization International Clinical Trials Registry Platform [http://www.who.int/ictrp/search/en/])** | **Result** |
|  | insomnia AND daridorexant | 26 |
| **set** | **Scopus** | **Result** |
| #1 | TITLE-ABS-KEY ( daridorexant ) | 273 |
| #2 | ( TITLE-ABS-KEY ( nemorexant ) OR TITLE-ABS-KEY ( act 541468 ) ) |  |
| #3 | #2 OR #1 | 279 |
| #4 | TITLE-ABS-KEY ( Sleep Initiation AND Maintenance Disorders ) | 18977 |
| #5 | ( TITLE-ABS-KEY ( DIMS ) OR TITLE-ABS-KEY ( Disorders of Initiating AND Maintaining Sleep ) OR TITLE-ABS-KEY ( Sleeplessness ) OR TITLE-ABS-KEY ( Insomnia* ) OR TITLE-ABS-KEY ( Chronic Insomnia ) OR TITLE-ABS-KEY ( Early Awakening ) OR TITLE-ABS-KEY ( Awakening , Early ) OR TITLE-ABS-KEY ( Nonorganic Insomnia ) OR TITLE-ABS-KEY ( Primary Insomnia ) OR TITLE-ABS-KEY ( Psychophysiological Insomnia ) OR TITLE-ABS-KEY ( Rebound Insomnia ) OR TITLE-ABS-KEY ( Secondary Insomnia ) OR TITLE-ABS-KEY ( Sleep Initiation* ) OR TITLE-ABS-KEY ( Dysfunction* , Sleep Initiation ) OR TITLE-ABS-KEY ( Transient Insomnia ) ) | 136,292 |
| #6 | #4 OR #5 | 136,292 |
| #7 | #3 AND #6 | 222 |

**Figure S1 Risk of bias and quality of evidence**

**
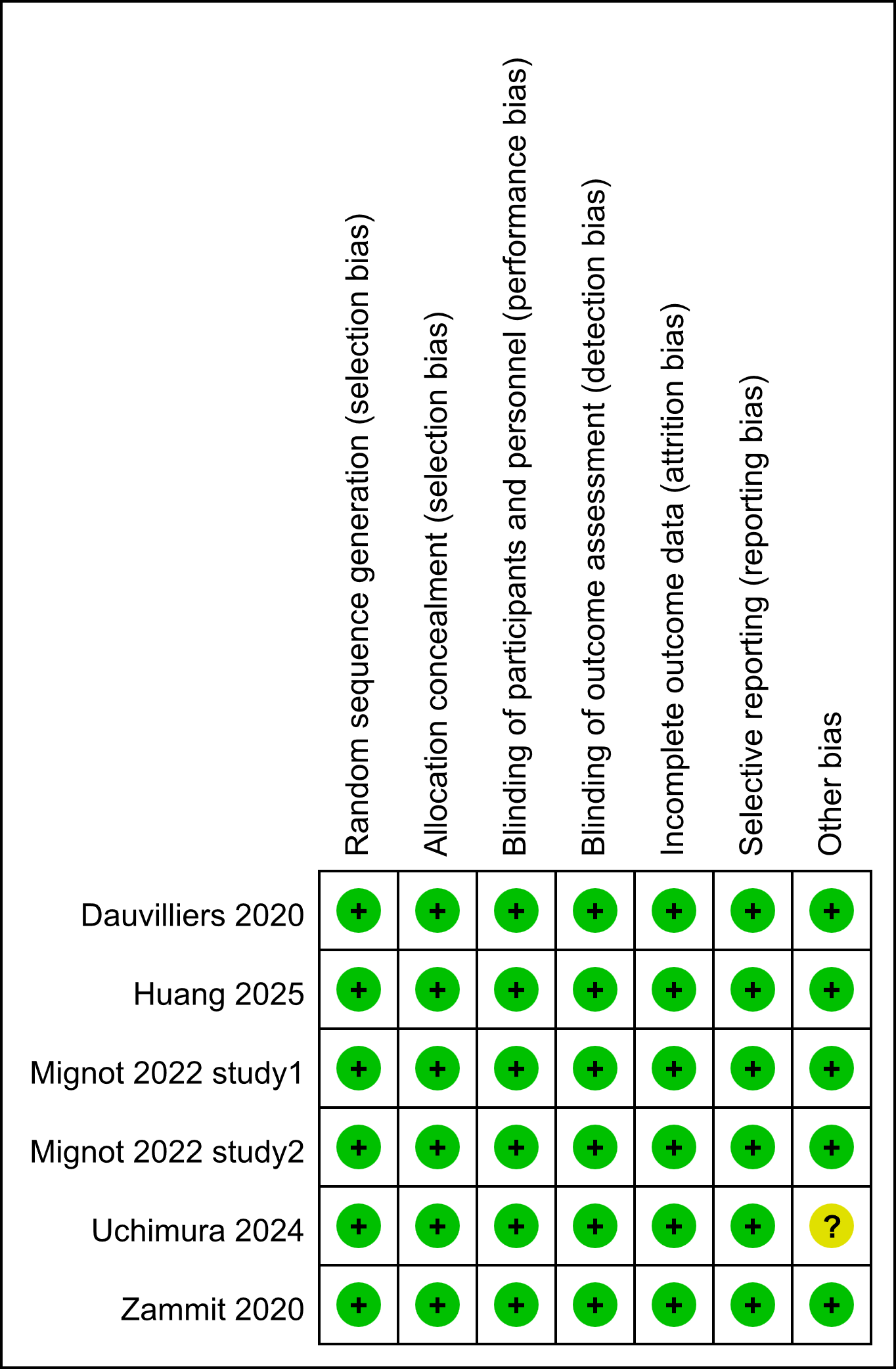
**

**Figure S2 sLSO at month 1**

**
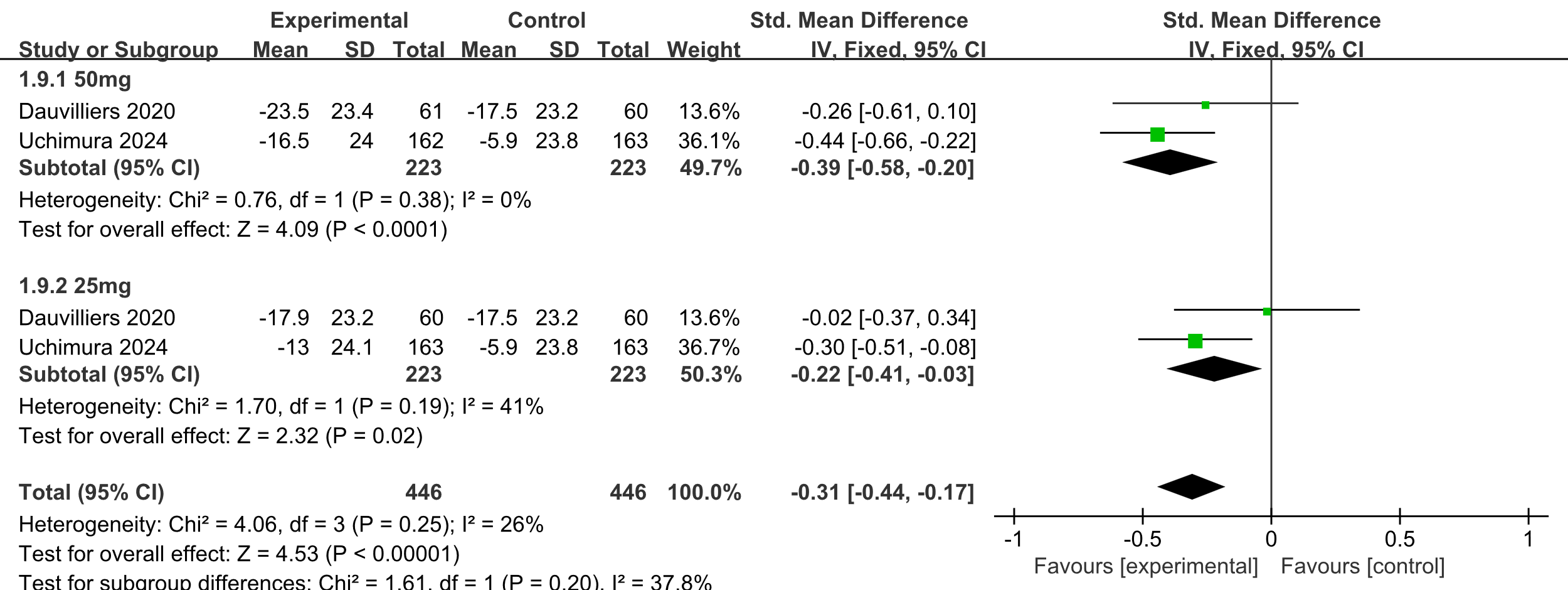
**

**Figure S3 sTST at month 1 and month 3**

**
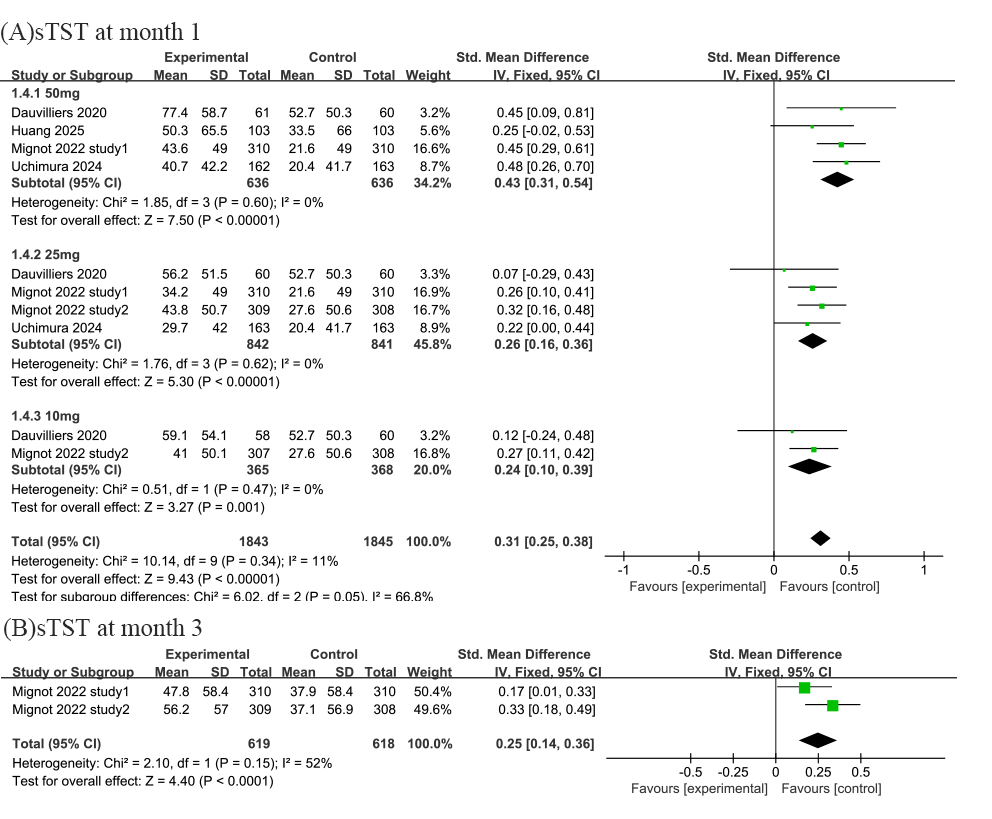
**

**Figure S4 The Visual analog scales (VAS) :** **quality of sleep at 4 week**

**
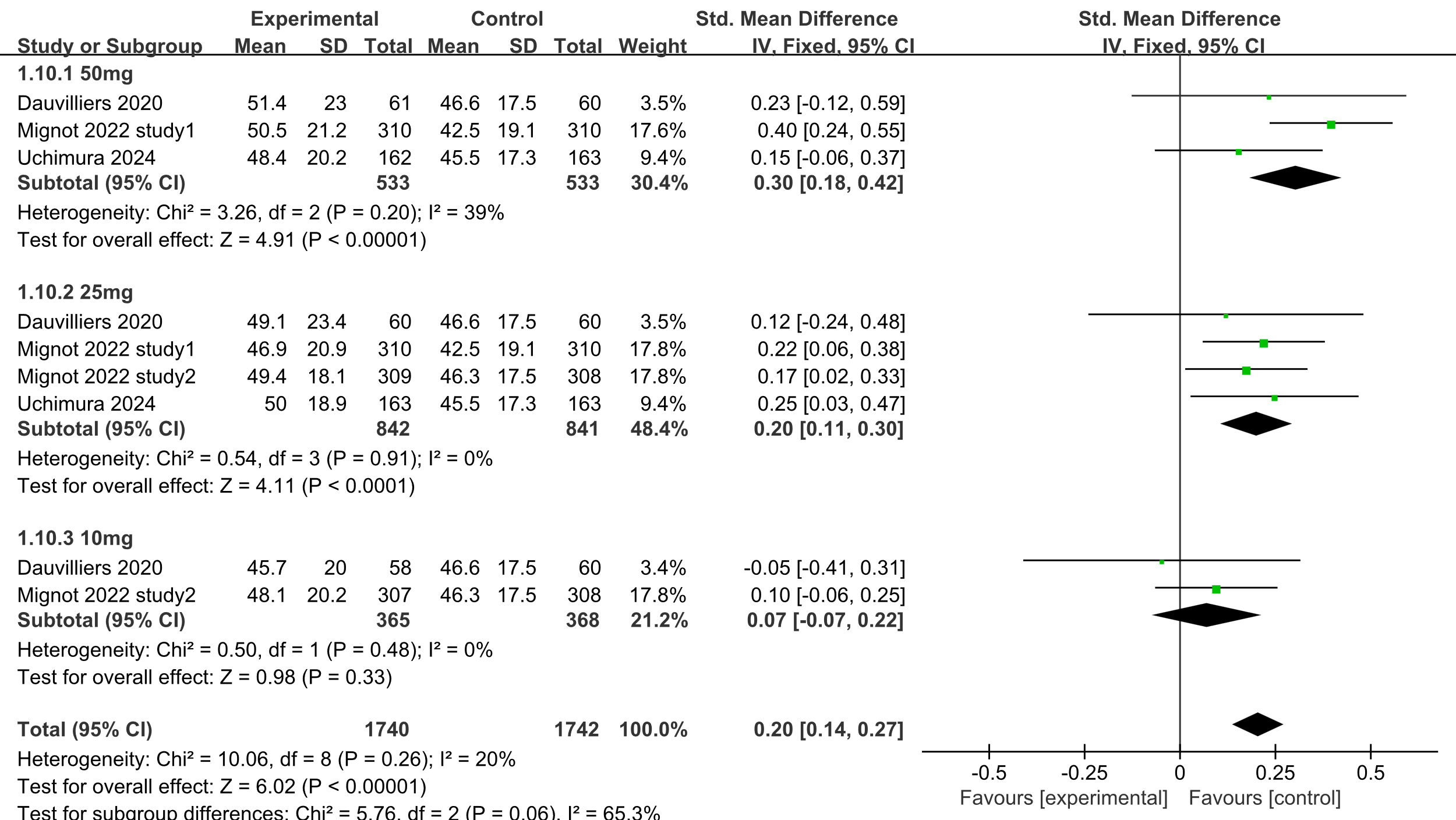
**

**Figure S5 The Visual analog scales (VAS) :** **depth of sleep at 4 week**

**
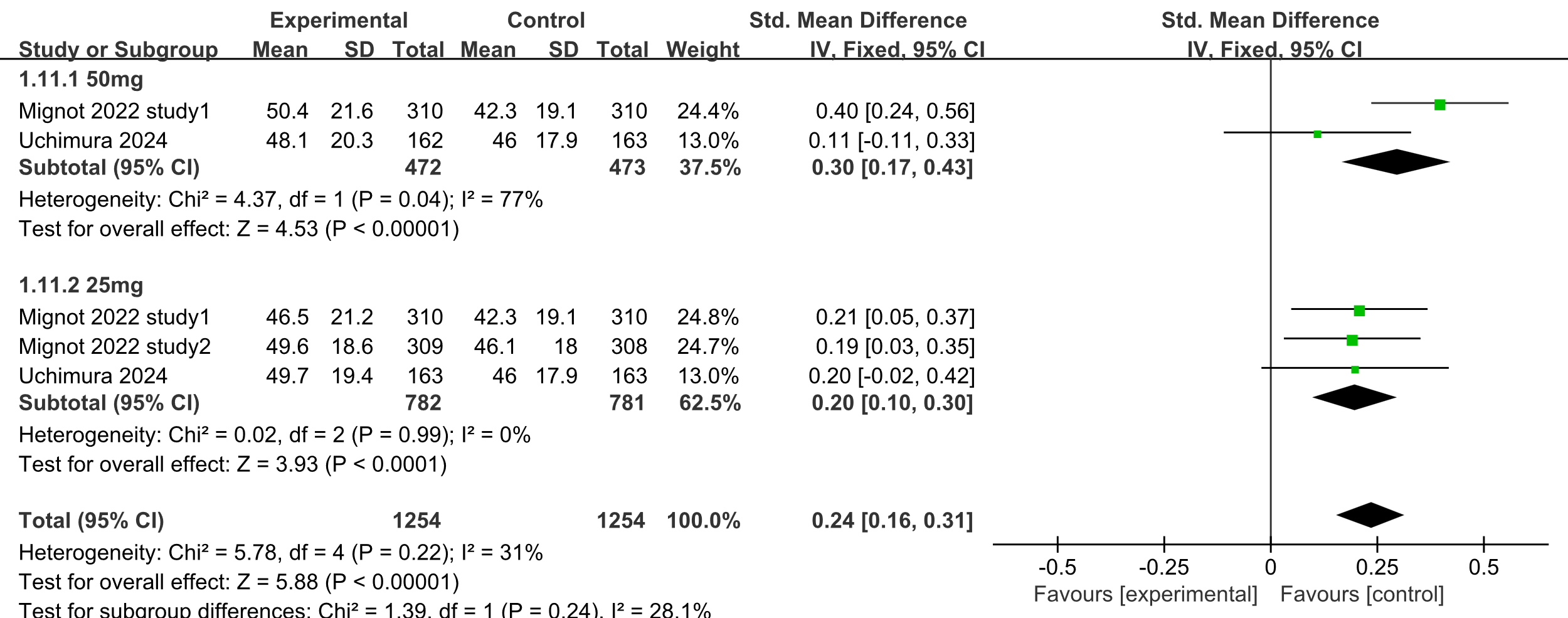
**

**Figure S6 The Visual analog scales (VAS) :** **daytime alertness at 4 week**

**
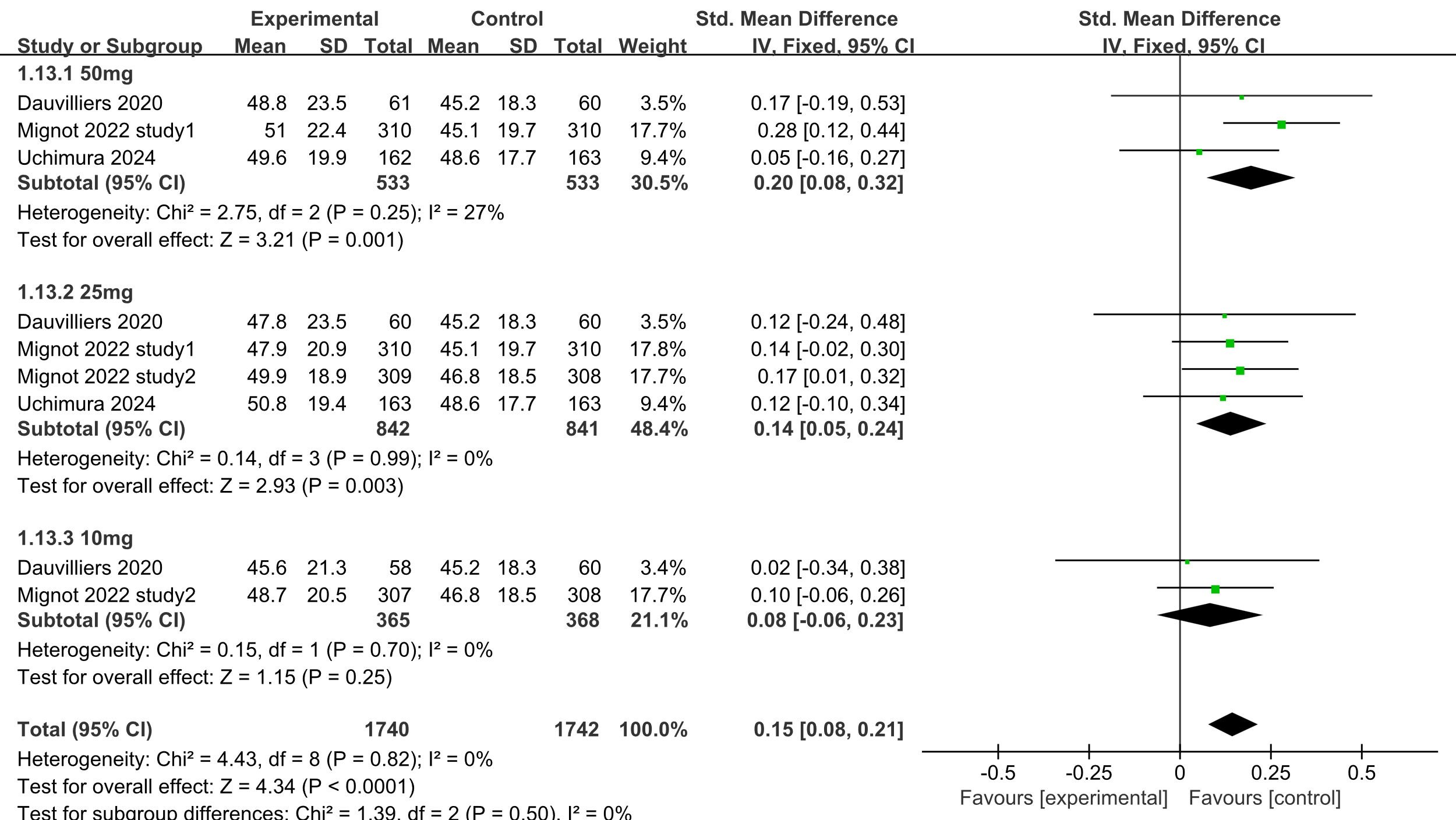
**

**Figure S7 The Visual analog scales (VAS) :** **ability to function at 4 week**

**
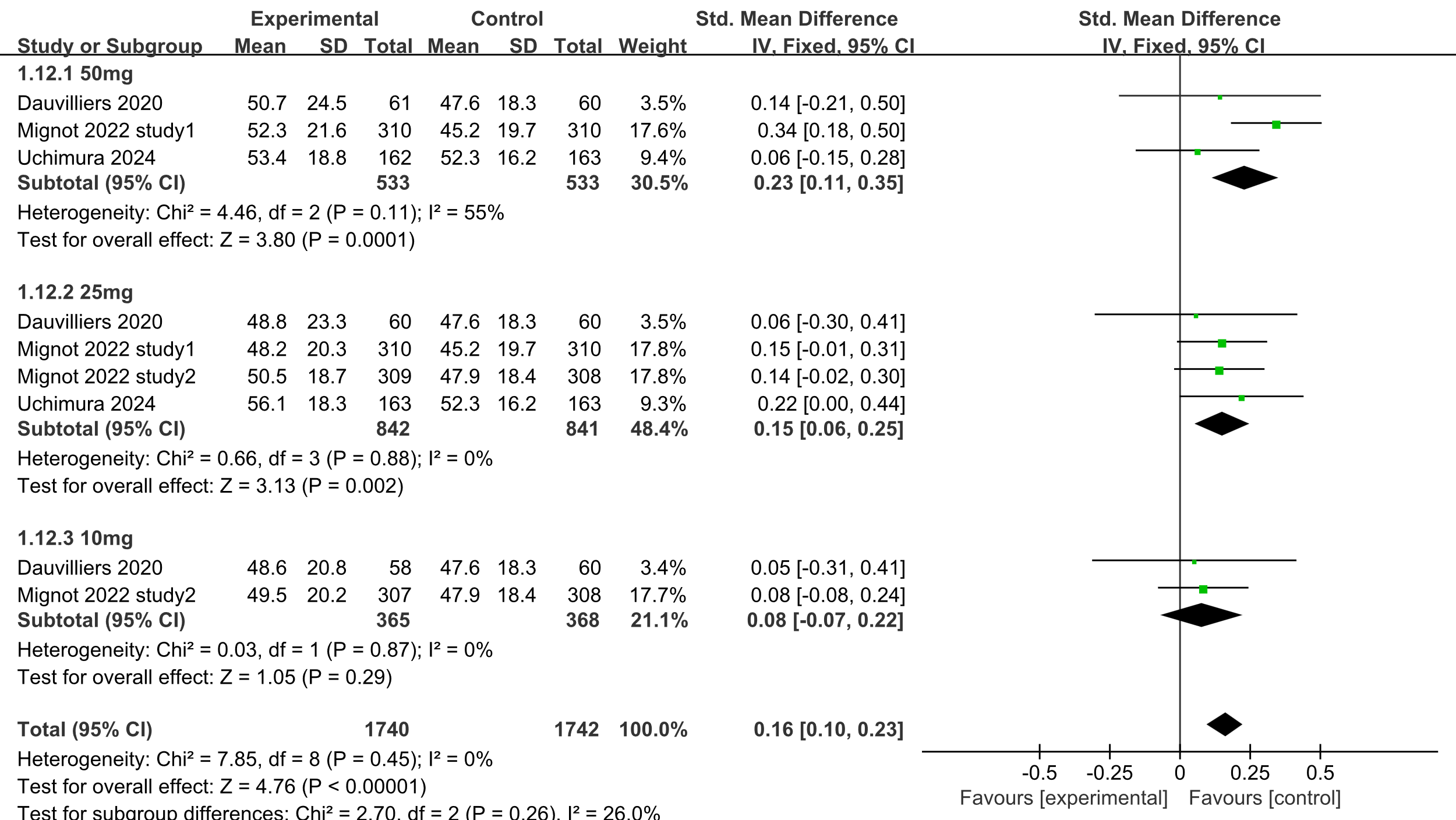
**

**Figure S8 IDSIQ sleepiness domain score at month 1 and month 3**

**
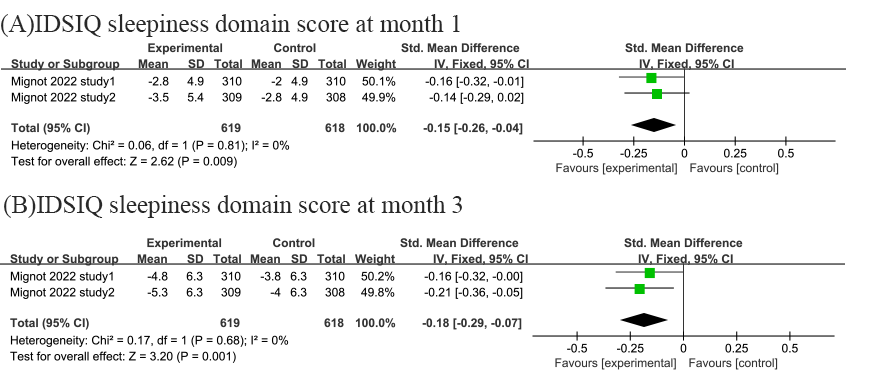
**

**Figure S9 Insomnia severity index (ISI) at 4 week**

**
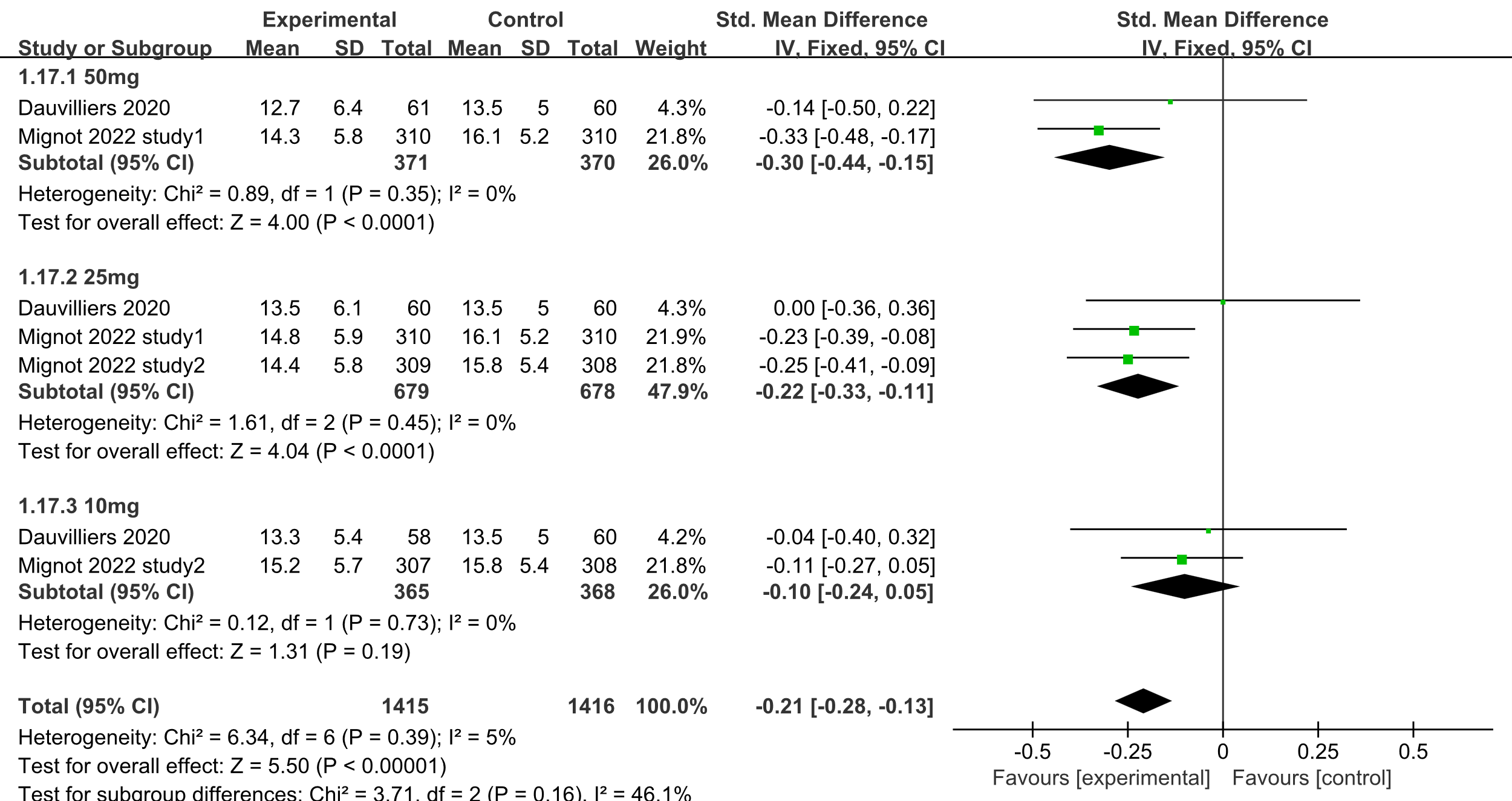
**

**Figure S10 Adverse events of nasopharyngitis**

**
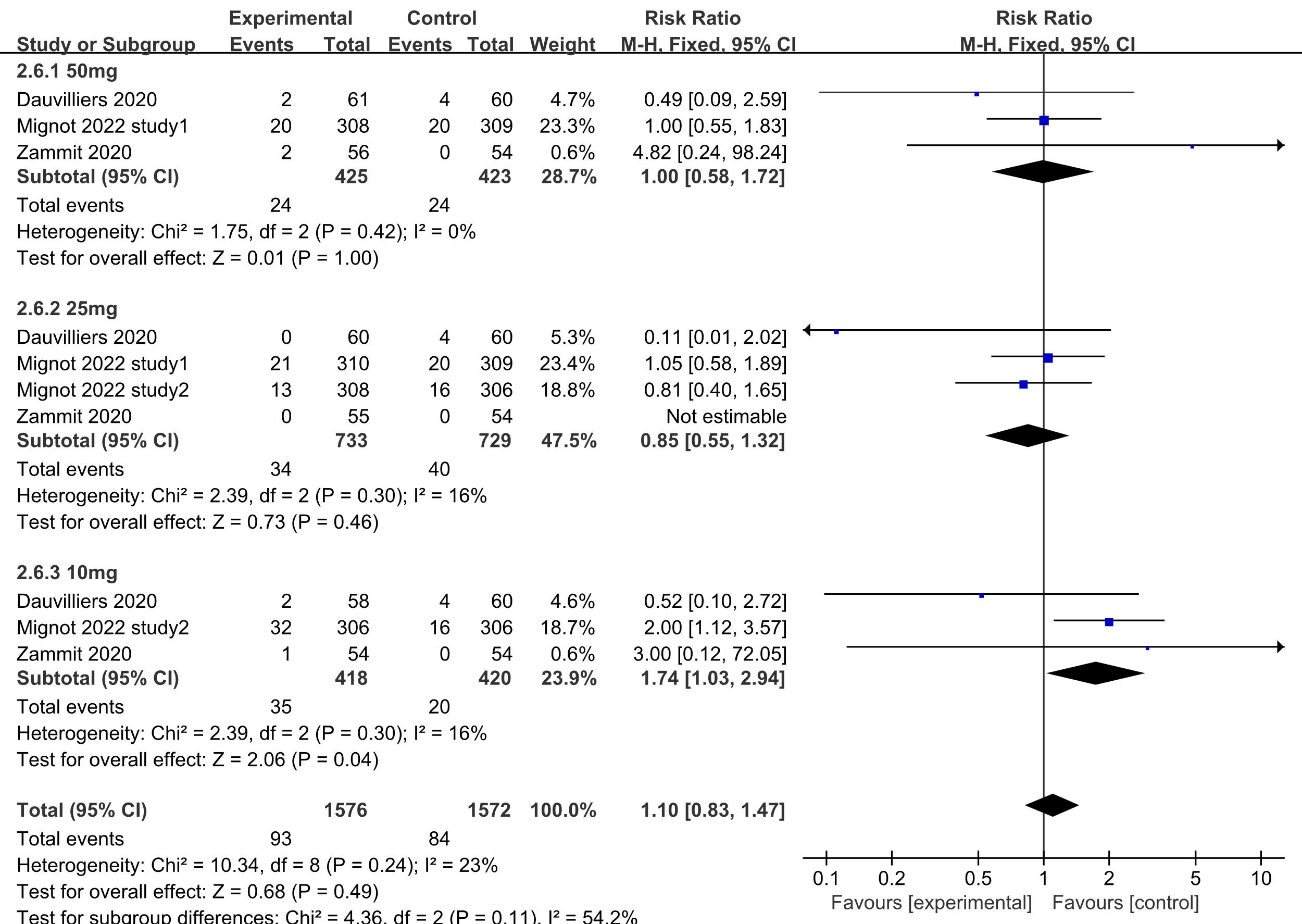
**

**Figure S11 Adverse events of headache**

**
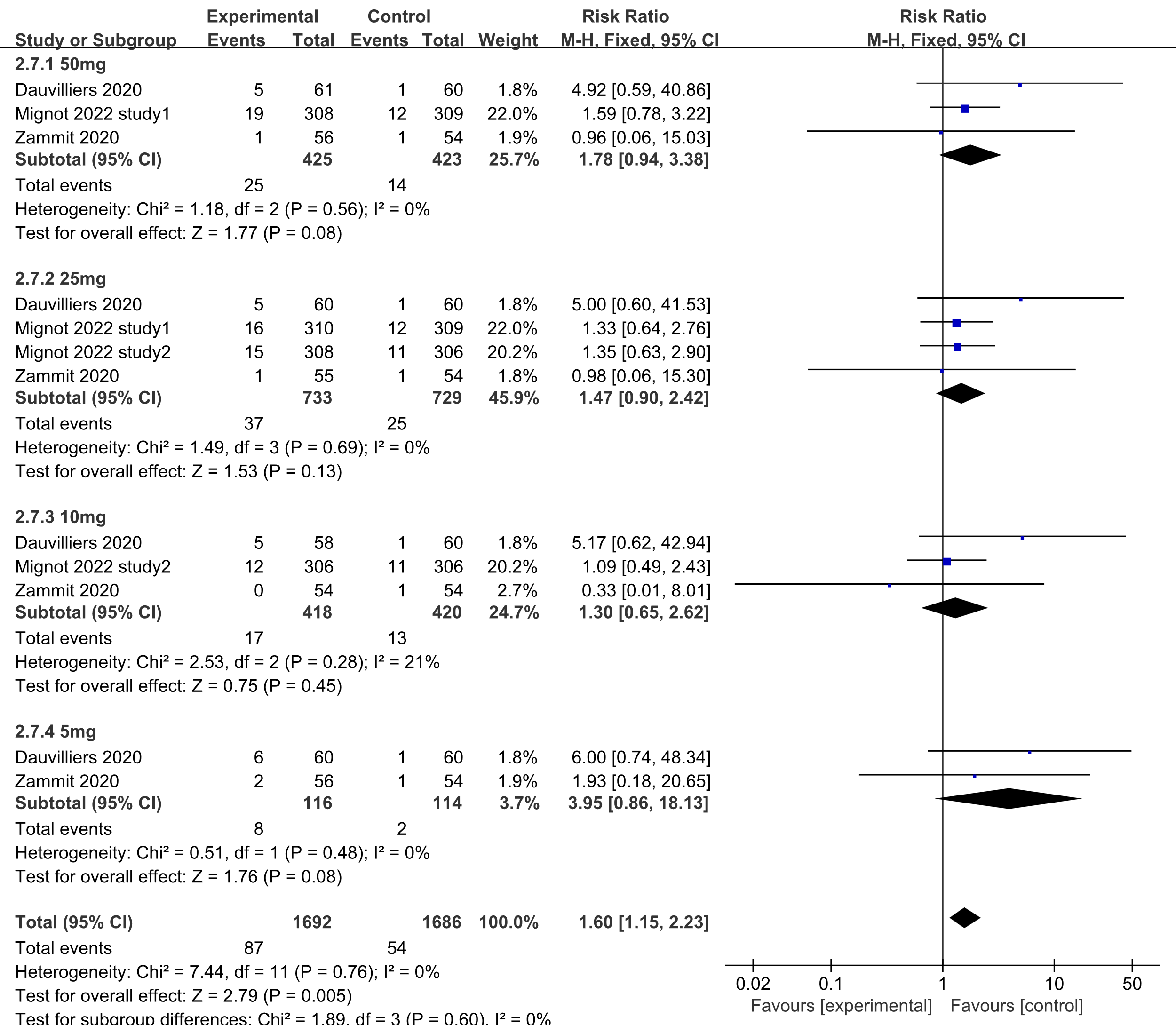
**

**Figure S12 Adverse events of somnolence**

**
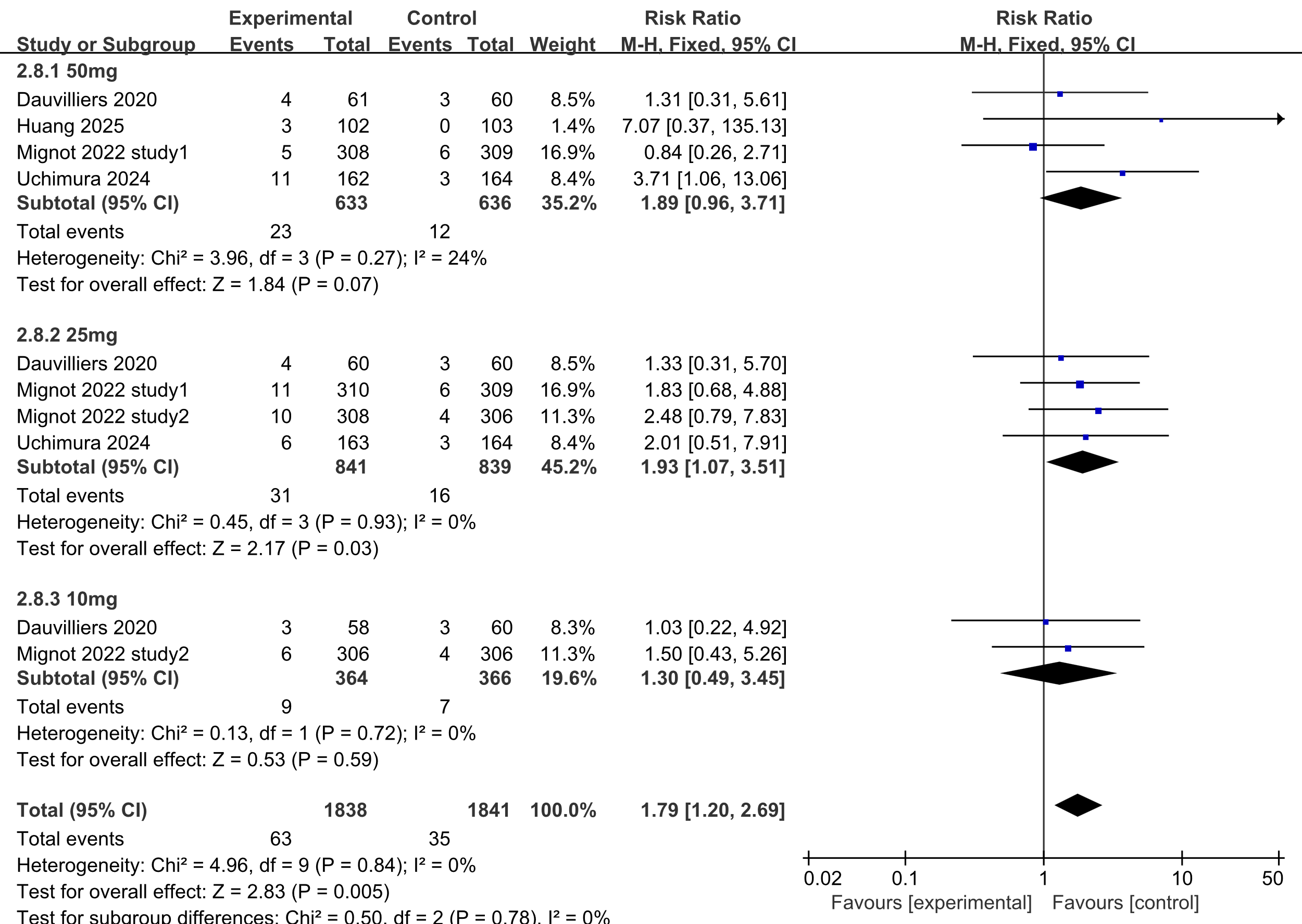
**

**Figure S13 Adverse events of fatigue**

**
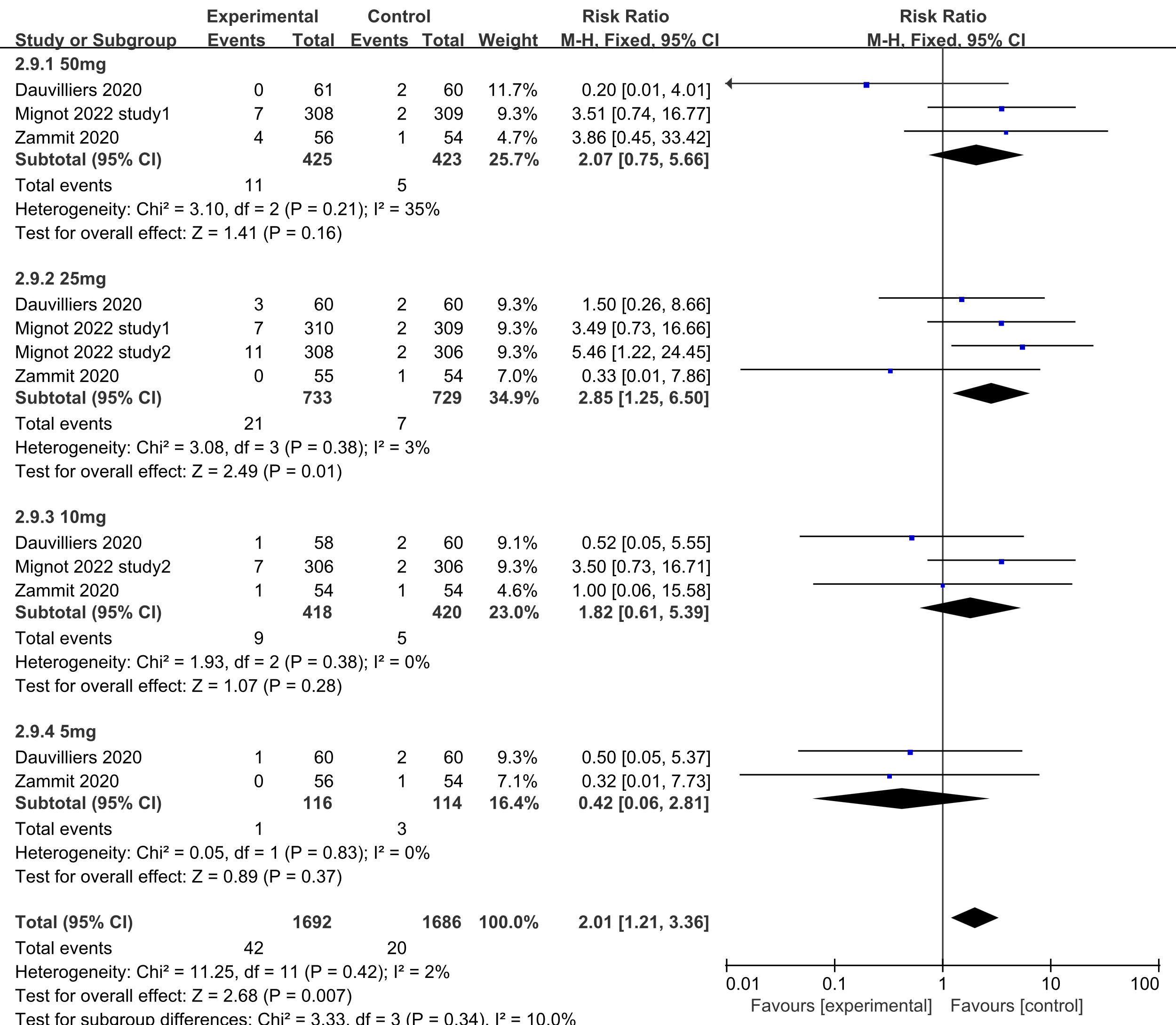
**

**Figure S14 Adverse events of dizziness**

**
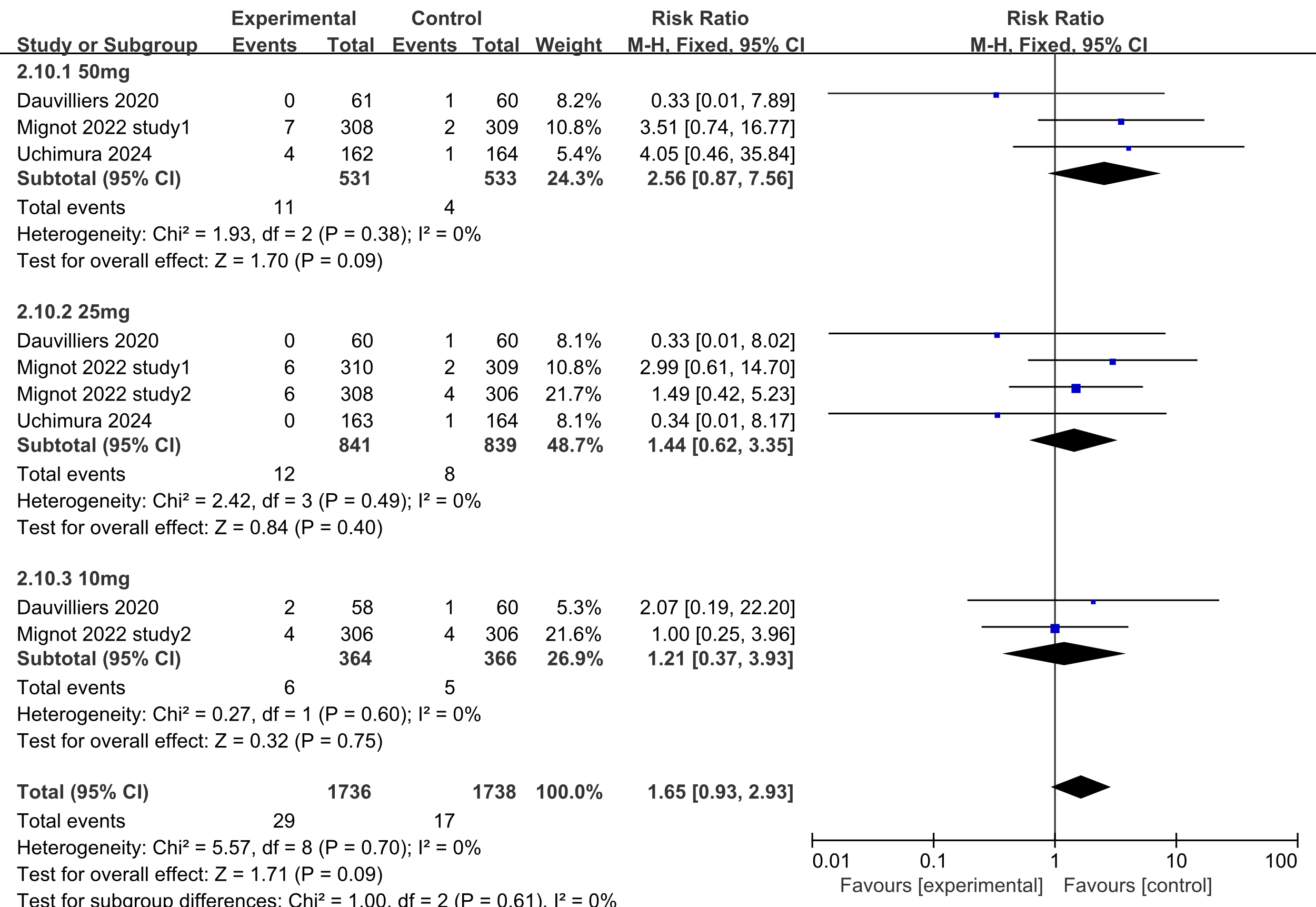
**

**Figure S15 Severe adverse events (SAEs)**

**
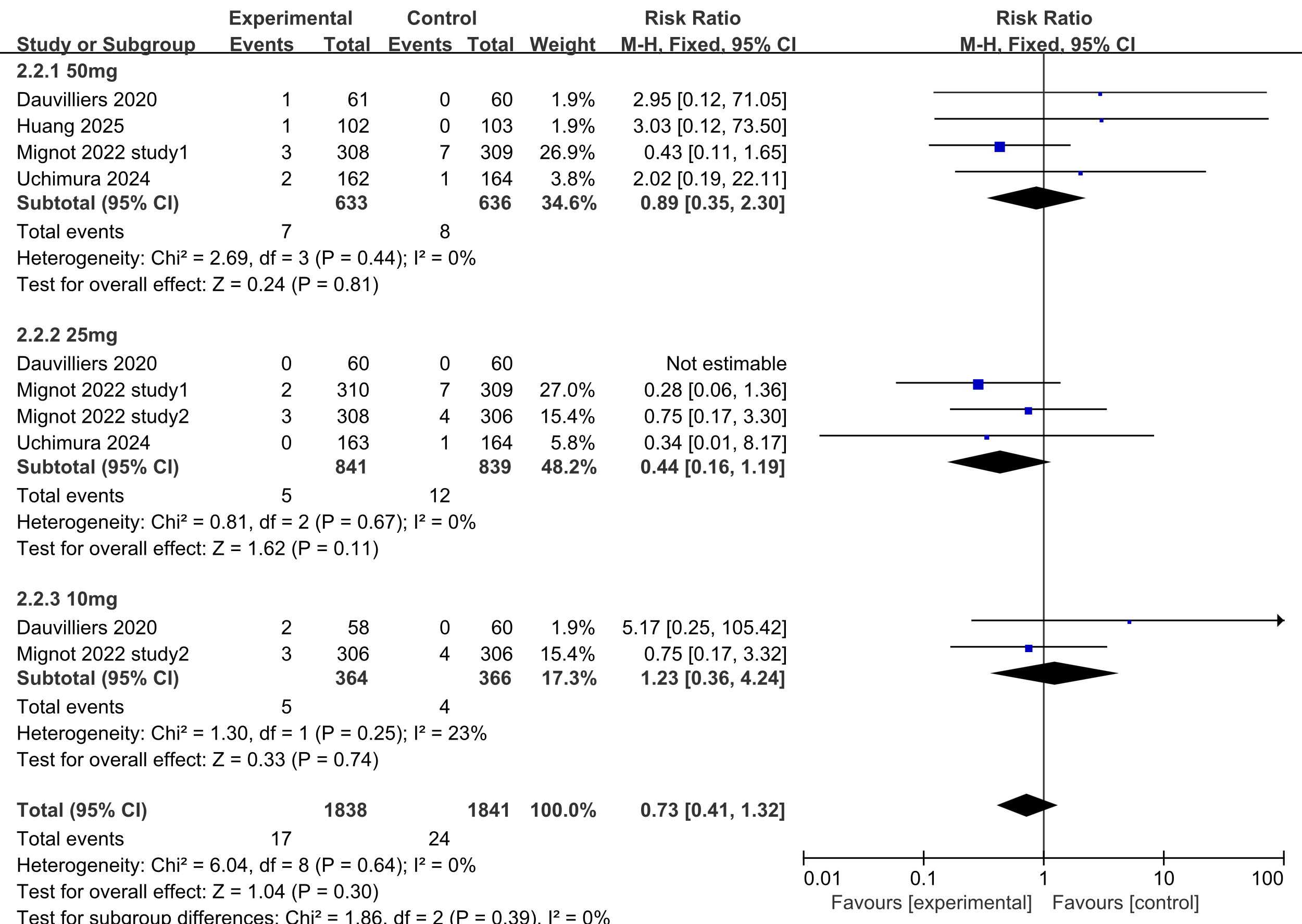
**

**Figure S16** **Adverse events leading to premature termination**

**
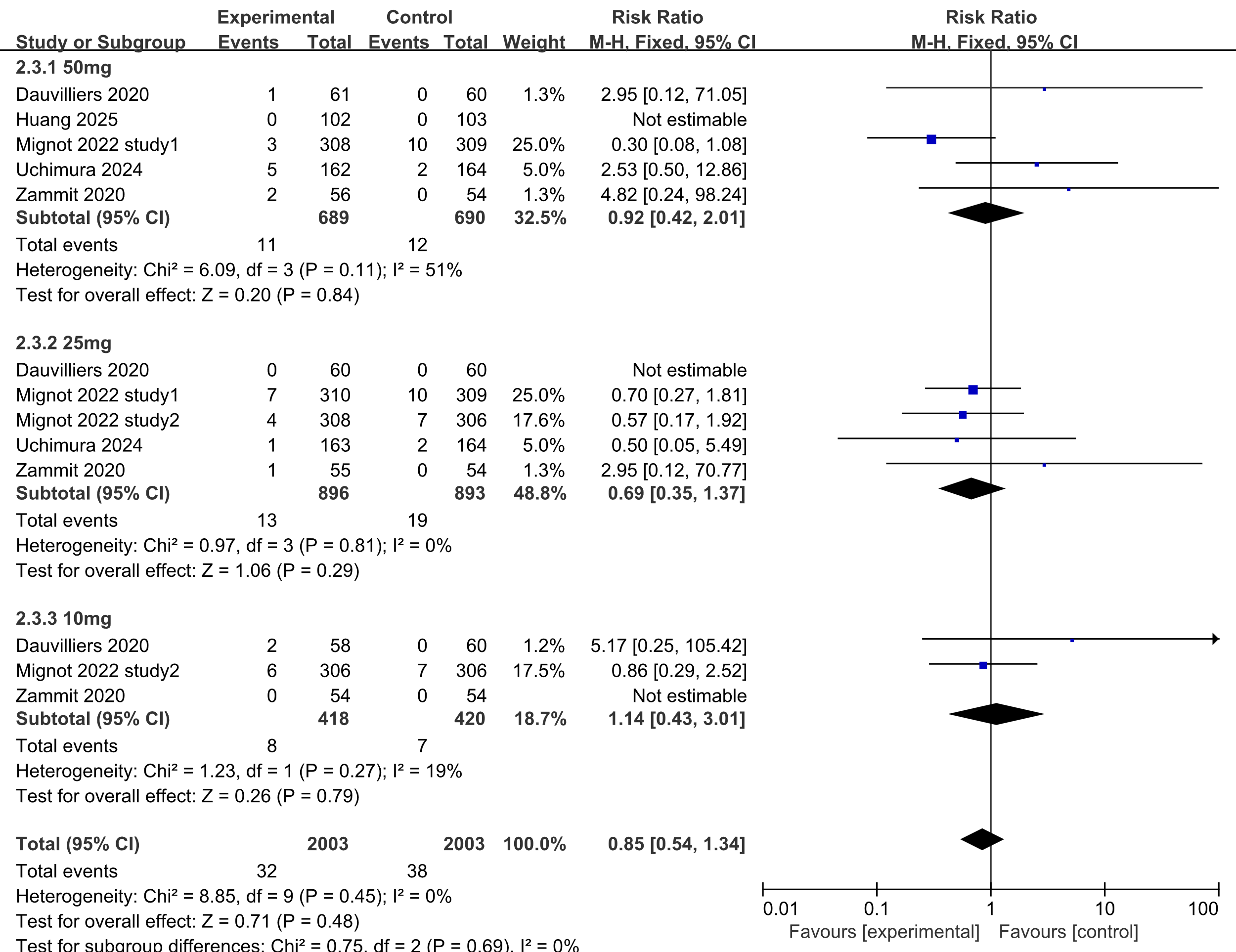
**

**Figure S17 Sleep paralysis**

**
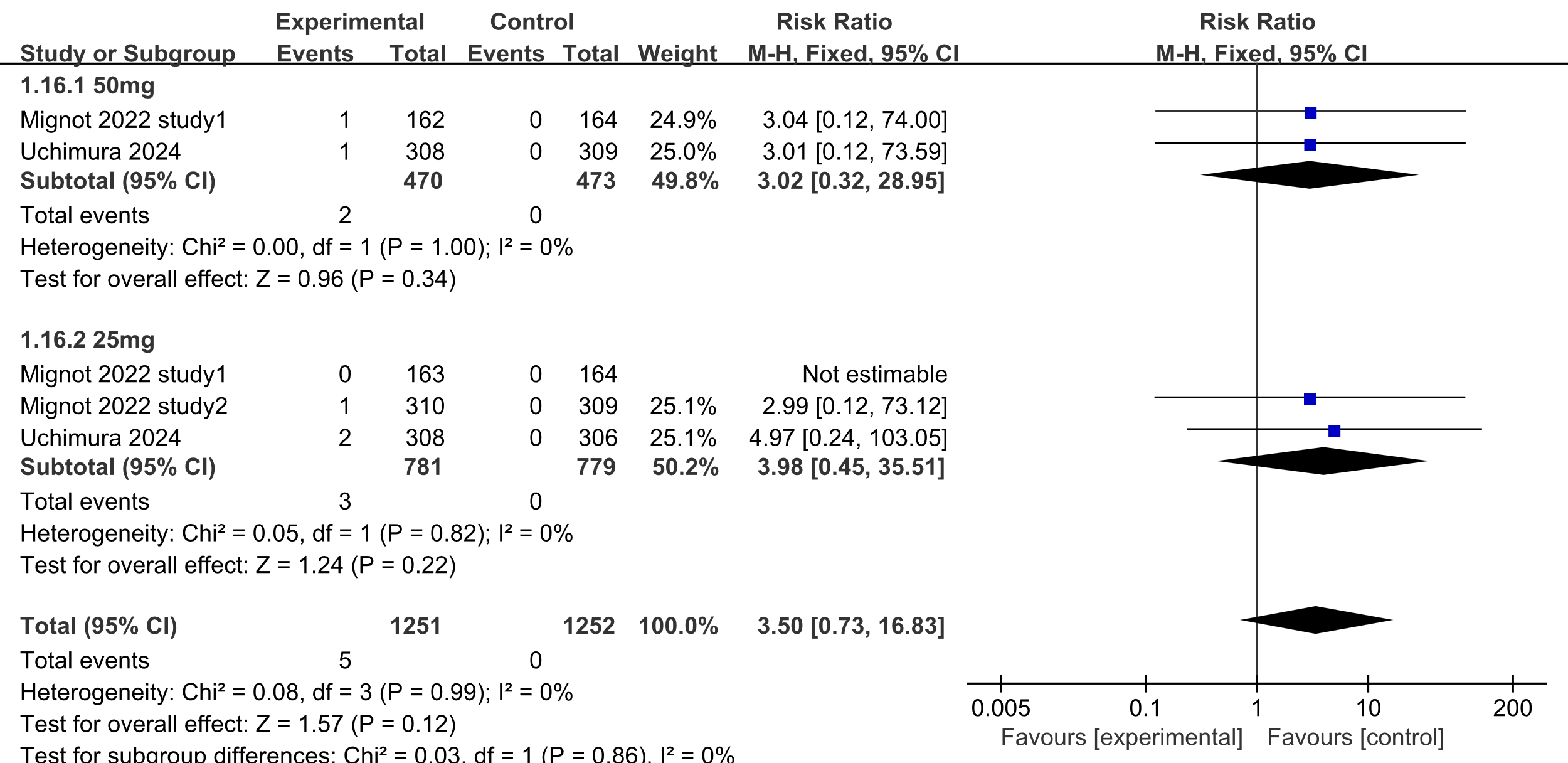
**

**Table S2 Distribution of Adverse Effects(The top 5)**

| Drugs  Adverse Effect | Placebo | Daridorexant  5mg | Daridorexant  10mg | Daridorexant 25mg | Daridorexant  50mg | Total |
| --- | --- | --- | --- | --- | --- | --- |
| Nasopharyngitis | 40 | 2 | 35 | 34 | 24 | 135 |
| Headache | 25 | 8 | 17 | 37 | 25 | 112 |
| Somnolence | 16 | 3 | 9 | 31 | 23 | 82 |
| Fatigue | 7 | 1 | 9 | 21 | 11 | 49 |
| Dizziness | 8 | 1 | 6 | 12 | 11 | 38 |
| Total | 96 | 15 | 76 | 135 | 94 | 416 |
